# Ventricular Assist Devices and Their Usage as a Bridge to Recovery in Patients With Acute Cardiotoxicity and Cardiomyopathy

**DOI:** 10.7759/cureus.10915

**Published:** 2020-10-12

**Authors:** Suyeewin Thiha, Chris A Robert, Abdul Rehman Z Zaidi

**Affiliations:** 1 Internal Medicine, University of Medicine (1), Yangon, MMR; 2 Obstetrics & Gynecology, Sunrise Hospital, Pune, IND; 3 Research Center, Dr. Sulaiman Al Habib Medical Group, Riyadh, SAU; 4 Medicine, Alfaisal University, Riyadh, SAU; 5 Medicine, King Fahad Medical City, Riyadh, SAU

**Keywords:** cardiotoxicity, cardiomyopathy, ventricular assist device, left ventricular assist device, myocarditis, lvad, heart failure with reduced ejection fraction, ejection fraction

## Abstract

Cardiotoxicity can reduce the heart’s function temporarily and is most commonly caused by radiation, immune reactions, and certain medications. Using a left ventricular assist device (LVAD) as a bridging therapy while waiting for cardiac recovery has been popular lately in patients who have a reduced ejection fraction after significant cardiac injury. Here we analyze the use of LVAD as a bridging therapy in three cases with chemotherapy-induced cardiotoxicity, acute myocarditis, and postpartum cardiac failure. Although LVADs are infamous for their device-related complications, the ejection fraction can increase up to 50% within days to months of usage without any complications in acute cardiotoxic patients that have no underlying significant risk factors or co-morbidities. Hence LVADs are excellent supportive devices while waiting for cardiac recovery, both in maintaining cardiac function and improving the associated organ failures.

## Introduction and background

Left ventricular assist devices (LVADs) have been well known lately for use as a bridge therapy while waiting for the transplant or as definitive therapy. These also have been a favorite choice for cardiologists while waiting for the cardiac recovery period in patients with heart failure with reduced ejection fraction (HFrEF) after significant cardiac injury [1].

Pathophysiology of cardiac injury and end-organ damage: Reduction of ejection fraction in cardiac injury can cause two significant outcomes: congestion and hypo-perfusion. Backward congestion due to incomplete left ventricular unloading can lead to pulmonary edema and subsequently increased right atrial pressure, and central venous congestion can lead to organ injuries especially renal, hepatic and gastro-intestinal injuries. Perfusion that is inadequate to meet the organ metabolic demands can lead to tissue hypoxia, cell injury, and ultimately organ failures. The significant organ failure according to hypovolemia includes acute kidney injury by reducing glomerular filtration rate, myocardial ischemic state, and stroke due to inadequate blood supply [2].

In this review, we focus mainly on acute cardiac injury both from medication and radiation but not limited to other significant causes. The patients included in this review have wide age variations from one year to 35 years of age with no prior risk factors or co-morbidities like diabetes mellitus or hypertension. English-only literature articles and full-text-only articles were reviewed for the sake of overcoming the language barrier in understanding the study and collecting more reliable information. We focused mainly on recent case reports which are less than five years old. However, the references found to be relevant were also assessed and reviewed even if they were greater than five years old to be included in this study.

A comprehensive search of PubMed was mainly performed due to its easy access, reliability, and resource validity in health-related articles. There are no ethical considerations while performing this study. The set of keywords we used are "heart assist device," "ejection fraction," "cardiomyopathic," and "cardiotoxicity." The purpose of this study is to review and evaluate the case reports relating to the effectiveness of LVADs as a temporary treatment in acute cardiotoxic and cardiomyopathic patients without underlying co-morbidities and significant risk factors for the sake of improving the overall health of the patients including end-organ damage while waiting for the recovery period.

## Review

We selectively used around 20-30 studies to assess full articles and nearly 13 relevant case reports were used to collect data in this literature review after using the specific set of keywords. We used English case-reports only to overcome the language barrier in understanding the studies and all the case reports published worldwide relating to the outcome and complication of LVADs used as a bridge to recovery in acute cardio-toxic and cardio-myopathic patients.

**Figure 1:**
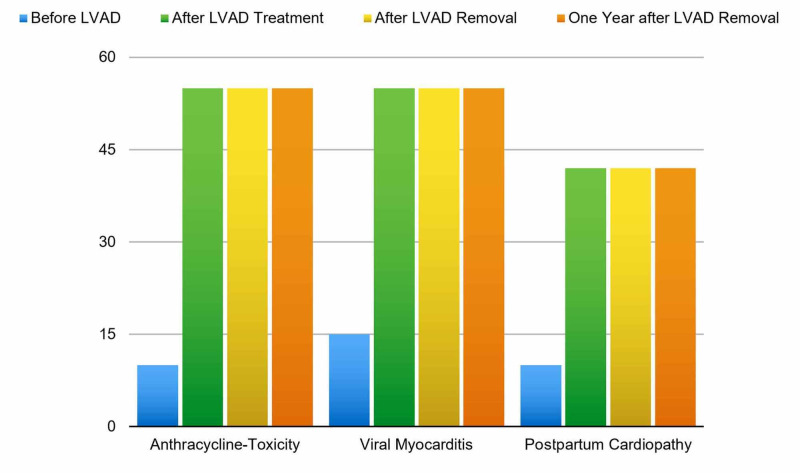
Ejection fraction percentages of patients with anthracycline toxicity, viral myocarditis, and postpartum cardiopathy LVAD = Left ventricular assist device

In this literature review, we focus mainly on any cardiovascular and device-related problems starting from one minute of post-LVAD treatment and its outcome on cardiac output while waiting for the cardiac recovery time. We have found that for patients with drug-induced cardiac toxicity or other cause of cardiac toxicity with no underlying significant risk factors or co-morbidities, the ejection fraction can increase nearly to 50% within days to months of usage and is stable during the one to two years follow up time without any cardiac complications (Figure 1).

We also found that its morbidity and mortality can be complicated with device-related problems including driveline infection as one of the most common complications followed by increase bleeding risk and cardiac tamponade as second, with pump thrombosis and pump failure subsequently as a third. Extremely rare but fatal complications include ventricular arrhythmia within 24 hours of post-LVAD treatment.

Case 1

A six-year-old girl with the diagnosis of acute myeloid leukemia had been treated with four cycles of chemotherapy including cytarabine, etoposide, daunorubicin, and mitoxantrone from December 2013 to April 2014. Serial echocardiogram throughout the chemo period showed 60-72% ejection fraction (EF) with normal left ventricular function. After completion of chemotherapy, her EF reduce to 44% with no symptoms. Despite that she was started on enalapril, furosemide, and milrinone. EF continued to reduce down to 21% on day nine of post-chemo and to <10% on day 25 with acute renal failure, respiratory failure, and acute hepatic failure. Right ventricular function was still normal at that time. After a multidisciplinary discussion, a left ventricular assist device (LVAD) was offered to this six-year-old girl with a cardiac index of 2.5-2.8 L/min, nitric oxide, unfractionated heparin, and aspirin. After 48 hours of LVAD, an echocardiogram showed right ventricular dilatation and dysfunction which required milrinone and low dose epinephrine as well as nitric oxide transition to tadalafil later for pulmonary vasodilatation. Despite these complications, there was an improvement in end-organ perfusion, and the patient recovered from respiratory failure after eight days of LVAD treatment. However, ectopic atrial tachycardia was found at the time and treated with amiodarone. After this initial period, the left ventricular ejection fraction (LVEF) becomes improved gradually with EF 55% on day 24 post-LVAD. Subsequently, LVAD was removed on day 29 and patient was discharged home on stable LVEF of 55-60% with normal end-organ functions under the control of multiple medications including aspirin, carvedilol, digoxin, enalapril, furosemide, spironolactone, tadalafil, levocarnitine, and ubiquinone. Follow-up for two years was done and no complications relating to cardiac function were reported [1].

Case 2

A 30-year-old woman without a significant past medical history and co-morbidities presented with tachycardia and dyspnea after one week of flu-like symptoms. Diagnosis of acute myocarditis was made and the patient was hospitalized. Despite maximal medication therapy, the patient became oliguric and her echocardiogram showed severe global hypokinesis with an ejection fraction of 15%. The patient was intubated on day two of hospitalization and an LVAD was offered. During LVAD installation, the patient became asystolic and resuscitation was done. She was successfully revived and LVAD was implanted with regular heparinization and anti-thrombin III therapy was done when the partial thromboplastin time decreased to less than 80. On day five of post-LVAD, platelet count became reduced to 46, and on day six and day nine of post-LVAD, clots on the interior blood-contacting surfaces of the right and left ventricular outflow tract were found which lead to changing of the blood pump. On day 14 of post-LVAD, bleeding from the cannulation site occurred. Despite these device-related complications, myocardial function gradually recovered and LVAD was removed on day 17 of post-LVAD treatment. Ten days after removal, the patient was discharged home with EF 45-50%. The patient was doing fine and no complications had occurred during seven months of follow-up [3].

Case 3

A 29-year-old left ventricular lady presented with symptoms of heart failure on the 36th week of gestation. Echocardiography showed a severely dilated left ventricle with end-diastolic diameter (EDD) 85 mm, and severely diminished global systolic function with a normal right ventricle. The mitral valve showed dilatation with grade II-III/IV regurgitation. The diagnosis of peripartum cardiomyopathy was made. A couple of days later, the delivery of the child through a cesarean section was done due to the worsening of heart failure. Then the patient was treated with dopamine, milrinone, noradrenalin, and intra-aortic balloon counterpulsation. Since there was no improvement despite maximal medication therapy, LVAD was offered. The patient was discharged home with LVAD and carvedilol, enalapril and aspirin after six weeks of post-LVAD treatment. She was placed on the waiting list for cardiac transplants because of no improvement in cardiac function after two months of LVAD treatment. Later, after two months, a gradual decrease in cardiac diameter and systolic function improvement was noticed on echocardiography follow-up. After nine months post-LVAD, the device was explanted and implantable cardioverter defibrillator​​​​​​​ (ICD) was placed prophylactically because of a large left ventricular apical scar. Within three years of follow-up, there were no heart-lung related complications with stable EF 42% and LVEDD 56 mm and no ICD shocks were given. Home medication included carvedilol 25 mg BID and enalapril 5 mg BID [4].

Discussion

By reviewing through recently published articles, we’ve found that using LVAD is like a double-edged sword. It can give us great benefit in maintaining cardiac index and end-organ perfusion in acute cardio-toxic patients. However, it also brings fatal adverse outcomes such as ventricular arrhythmia as a rare but the most serious complications within 24 hours of post-LVAD treatment, cardiac tamponade, secondary device-related infections, GI bleeding, and pump failure. Without anti-coagulation, pump thrombosis followed by life-threatening pump failure is also the most common device-related outcome within a short period after post-LVAD treatment. However, with the help of anti-coagulants, we can overcome this adverse effect in temporary LVAD treatment.

Because of the above complication risks, it is crucial for temporary LVAD treatment patients not only to be treated with anti-coagulants to prevent pump thrombosis but also to be monitored regularly to prevent fatal adverse outcomes (Table 1) [5-12].

**Table 1:** Left ventricular assisted device-related complications in acute cardiotoxic patients GI = gastrointestinal

Serial Number	Complications	Duration
1	Cardiac Tamponade	Within days
2	Device Related Infections	Within months to years
3	GI Bleeding	Within months to years
4	Thrombosis	Within months to years
5	Arrhythmia	Within days
6	Pump Failure	Within months to years

The majority of patients only used LVAD for a short time while waiting for the cardiac recovery period, commonly under one month. Nevertheless, there are several acute cardiotoxic patients without underlying cardiac risk factors who have to use LVAD for more than one month. There is also no interpretation of data on quality of life (QOL) score while reviewing several case reports using PubMed and Google Scholar. Theoretically, we can expect the QOL score to be easily reduced according to its device-related complications and multiple post-LVAD treatments. But, for the acute cardiotoxic patients who used LVAD only for a short period, commonly under one to three months, the QOL score would not be much affected [1,3,4,13-15].

Regarding the pathophysiology of the cardiac function relating to other organs, mainly lungs, kidney, liver, and brain, maintaining the adequate ejection fraction while waiting for the cardiac recovery is another important factor to control as reducing cardiac function can subsequently lead to irreversible failure of related organs. That is why by maintaining the cardiac function, LVAD can protect not only the backward congestion with pulmonary edema, subsequent right ventricular failure, and organ injuries including renal, hepatic, and gastrointestinal injuries but also subsequent inadequate perfusion and tissue hypoxia which can lead to acute kidney injury, stroke and myocardial ischemic state in addition to the underlying toxic myocardium [13,16].

In summary, in this literature review on the treatment of acute cardio-toxic and cardiomyopathic patients using LVADs, focused mainly on case reports, we found out that using an LVAD can contribute to an overall improvement in cardiac performance and protecting the end organs from damage; however, it is accompanied with multiple adverse effects including life-threatening conditions. Nevertheless, after comparing and contrasting of multiple case reports including its benefits and complications, we propose that LVADs are currently an excellent device to maintain the adequate cardiac output and protecting the other organs while waiting for the cardiac recovery time as long as we can overcome its device-related complications with careful monitoring and additional medical treatments [1,3,4,13,14,17-19].

## Conclusions

Although LVADs are notorious for their significant morbidity and mortality relating to its device-related complications including bleeding (e.g. cardiac tamponade and GI bleeding), thrombosis, arrhythmia and pump failure which can lead to sudden death within days of post-LVAD treatment, these factors can be overcome by using medication support, serial post-LVAD investigations and close follow up. Though there are not enough data relating to the QOL score, we can conclude that the quality of life of the patients will not be much effective as it is only used as a temporary treatment. However, several important decisions must be made before placing an LVAD in a patient with an acute cardiotoxic state including choosing between cardiac transplant and LVAD treatment. Moreover to choosing the appropriate devices, we can conclude that ventricular assist devices are excellent supportive devices during the cardiac recovery period in maintaining the cardiac function in addition to protecting and improving potential associated organ failures.
